# Effects of Different Shaping Methods and Loading on Fruit Quality and Volatile Compounds in ‘Beibinghong’ Grapes

**DOI:** 10.3390/foods14050772

**Published:** 2025-02-24

**Authors:** Yingxue Liu, Weiyu Cao, Baoxiang Zhang, Hongyan Qin, Yanli Wang, Yiming Yang, Peilei Xu, Yue Wang, Shutian Fan, Changyu Li, Jiaqi Li, Wenpeng Lu

**Affiliations:** 1 Institute of Special Animal and Plant Sciences, Chinese Academy of Agricultural Sciences, Changchun 130112, China; liuyingxue82@163.com (Y.L.); 82101231147@caas.cn (W.C.); zhangbaoxiang@caas.cn (B.Z.); qinhongyan@caas.cn (H.Q.); wangyanli@caas.cn (Y.W.); yangyiming@caas.cn (Y.Y.); xupeilei@caas.cn (P.X.); wangyue05@caas.cn (Y.W.); fanshutian@caas.cn (S.F.); lichangyu@caas.cn (C.L.); lijiaqi@caas.cn (J.L.); 2 College of Horticulture and Landscape Architecture, Heilongjiang Bayi Agricultural University, Daqing 163319, China

**Keywords:** tree shape, load capacity, yield, quality, metabolome, volatile metabolites

## Abstract

The effects of different shaping methods and loading treatments on the photosynthetic rate, chlorophyll content, fruit yield and quality, and volatile compound composition of the ‘Beibinghong’ grape were studied. In the experiment, 6-year-old ‘Beibinghong’ grapes were selected as the material, and two kinds of shaping methods were adopted: the double main vine upright tree (control) and the inclined horizontal dragon tree. The inclined horizontal dragon tree was treated with different loads. The volatile components in grapes were analyzed by gas chromatography–mass spectrometry (GC-MS). The changes in quality and volatile components of ‘Beibinghong’ grape under different treatments were analyzed by multivariate statistics. The results showed that the inclined horizontal dragon tree significantly increased the net photosynthetic rate and chlorophyll content of leaves, and increased the soluble sugar content and sugar–acid ratio of fruits. The quality of grapes was better than that of the upright tree with double main vine. The results of loading showed that the plants with nine fruit branches had higher net photosynthetic rate and yield, and the best performance in reducing sugar content, titrable acid content and sugar–acid ratio, which was the most suitable loading treatment. The results of metabolomics study showed that 291 volatile metabolites were identified, of which 25 were considered to be the key differential metabolites affecting the flavor of ‘Beibing red’ fruit under different treatments. Further analysis showed that the inclined horizontal draconite tree was superior to the double main draconite tree in improving fruit quality and accumulation of volatile compounds in fruit. This study revealed the regulation mechanism of different shaping methods and loading loads on the growth and fruit quality of ‘Beibinghong’ grapes, which provided theoretical support for optimizing the viticulture management of ‘Beibinghong’ and improving the fruit quality and market competitiveness.

## 1. Introduction

The grape (*Vitis vinifera* L.) is one of the most widely cultivated fruit trees in the world [1,2]. With the increasing demand for wine, the quality of wine grapes and wine has become a focus of attention. The planting of wine grapes in China started long ago in the Western Han Dynasty, but it developed rapidly in the late 1970s and early 1980s [3]. Wine grapes have rich nutritional value, are an important raw material of wine production, and their quality plays a decisive role in the quality of wine. The growth and development process of wine grapes is regulated by many factors. Also, there are many factors affecting fruit quality, such as soil conditions, temperature, light [4], water and fertilizer, pests and diseases, hormone application [5], management methods, etc.

In the process of viticulture, different shaping and loading methods have significant effects on plant growth and development and fruit quality [6]. Different shaping methods will lead to differences in total leaf area, light energy utilization of branches and vines, fruit bearing characteristics and susceptibility to diseases, and pests of wine vines [7]. Shaping methods directly affect the light distribution, ventilation, light transmission conditions, and nutrient distribution of plants, which then affect the fruit yield and quality. Different shaping methods have a significant impact on the fruit quality of wine grapes [8,9,10]. On the other hand, load is a key factor in regulating the balance of plant reproduction and vegetative growth. A reasonable load can not only improve the fruit yield, but also improve the internal quality of the fruit, such as the sugar–acid ratio, aroma composition, etc., which is a necessary measure to obtain high yield and quality of grapes [11,12]. Load regulation is an important means of regulating the relationship between fruit growth and fruit material transport and distribution. It effectively solves the contradiction between vegetative growth and reproductive growth [13,14]. Improving grape quality through reasonable loads is a simple and feasible method, which can improve the microenvironment of wine grape growth to varying degrees. It can assist grapes in reaching the ideal maturity, meeting the target yield, and reducing nutrient consumption, and thus obtain higher yield and excellent quality [15].

The ‘Beibinghong’ grape is a unique mountain grape variety in China, mainly distributed in northeast China [16,17]. Because of its unique cold resistance and flavor characteristics, it has gradually attracted the attention of researchers and wine producers in recent years. However, there are few studies on the effects of different shaping methods and load treatment on the fruit quality and volatile matter of the ‘Beibinghong’ grape. Based on the previous research, we speculated that different tree shape and load capacity would have a great impact on the yield, quality, and wine quality of the Beibing red grape. In this study, the ‘Beibinghong’ grape was used as the experimental material. By comparing the difference between the upright tree shape and the inclined horizontal tree shape, the shaping of the double main vine, the setting of different load treatments, the effects of different shaping methods and loads on the leaf photosynthetic rate, chlorophyll content, fruit yield, quality, and volatile compounds of the ‘Beibinghong’ grape were systematically studied. By measuring the metabolomics of fruit volatile compounds, multivariate statistical analysis was adopted to comprehensively analyze the changes in quality and volatile components of the ‘Beibinghong’ grape under different tree shape and load conditions, and screen key metabolites affecting fruit flavor from multiple dimensions. In order to reveal the regulation mechanism of different shaping methods and load on the growth and fruit quality of the ‘Beibinghong’ grape, we finally determine the appropriate shaping methods and reasonable load and provide a scientific basis for its production and cultivation.

## 2. Materials and Methods

### 2.1. Experimental Materials and Treatments

The sampling site was located in the national fruit germplasm mountain grape nursery of Zuojia Town, Jilin City, Jilin Province (longitude 126°08′20″, longitude 44°06′82″). The experimental materials were selected from the 6-year-old mountain grape variety ‘Beibinghong’, which was planted along the east–west line with a row spacing of 1.0 m × 2.5 m. The management of soil, fertilizer, water, and pest control in the field was carried out in accordance with conventional methods. During the period from June to August, at 9 a.m. on sunny days, the net photosynthetic rate and chlorophyll content of the first leaf blade of the 1st to 4th leaves on each plant were measured. The net photosynthetic rate was determined using the CIRAS-2 photosynthesis analyzer (PP·SYSTEMS of Lufthansa Company in the Hitchin, UK), and each treatment was conducted on 6 trees. The chlorophyll content was measured using the CCM-200 chlorophyll analyzer, with 3 leaf blades measured per tree, and 6 trees were measured for each treatment. For each treatment, the yield of each tree was measured individually. Thirty grains were selected for single-grain weight measurement in each treatment, and then the fruit was squeezed to extract juice. The soluble sugar, titratable acid and tannin contents were determined using the Perrin reagent method, the NaOH titration method and the Folin–Denis method, respectively. In addition, samples of all fruits under different treatments were collected for wine-making tests to determine the metabolomics test. The wine-making method adopted the conventional wine-making process.

The experiment used the vertical tree shape of double main vine, commonly used in production, as the control (L1), and the inclined horizontal dragon trunk tree as the treatment. In order to determine the reasonable loading capacity of the inclined horizontal dracake tree, five loading treatments (L2–L6) were set up, with the fan-shaped leaf curtain shape for the upright double-tensioned tree and the vertical leaf curtain shape for the inclined horizontal dracake tree. There were three replicates per treatment and six trees per replicate. See Table 1 for specific test treatment and the amount of retained branches and fruits.

### 2.2. Detection Methods of Basic Physical and Chemical Indexes

When the ‘Beibinghong’ grapes were ripe, the harvest was carried out and the yield per plant was measured. Six plants were measured per treatment. The content of titrable acid in grape juice was determined with reference to the indicator method in GB/T 15038-2006 [18], which is the analytical methods of wine and fruit wine. According to the principle of acid-based titration, phenolphthalide was used as indicator and titrated with standard alkali solution [19,20]. The total sugar content in grape juice was determined by anthrone sulfuric acid colorimetric method, and the standard curve was drawn by standard glucose solution. The tannin content in grape juice was determined by the Folin–Denis method. The blue compounds were formed by reaction of tannin and phosphopolybdic acid soaked in sodium carbonate solution at 85 °C for 3 h. Absorbance was measured at 740 nm. The net photosynthetic rate was measured according to Zhang Weiyuan’s [21] method, and each plant was measured three times in parallel, and the average value was taken to determine 6 plants. The relative chlorophyll content of the 2nd~4th leaves of ‘Beibinghong’ grape bud was measured by CCM-200 chlorophyll analyzer. The average value was repeated 30 times, and the results were expressed by chlorophyll content index (CCI).

### 2.3. Sample Preparation and Extraction for Widely Targeted Metabolome Analysis

Transfer the sample to a 50 mL centrifuge tube and freeze-dry it. Grind the freeze-dried sample into a powder, weigh 30 mg of the sample, and place it into a 1.5 mL centrifuge tube. Add two small steel balls and 600 μL of methanol–water (*V*:*V* = 1:1, containing mixed internal standards, 4 μg/mL). Pre-cool in a −40 °C freezer for 2 min, then grind in a grinder (60 Hz, 2 min). Perform ultrasonic extraction in an ice-water bath for 30 min. Add 150 μL of chloroform and vortex for 2 min. Perform ultrasonic extraction in an ice-water bath for 30 min. Let it stand at −40 °C for 30 min. Centrifuge at low temperature for 10 min (12,000 rpm, 4 °C), and transfer 30 μL of the supernatant to a glass derivatization vial. Evaporate the sample to dryness using a centrifugal concentrator. Add 80 μL of methoxyamine hydrochloride pyridine solution (15 mg/mL) to the glass derivatization vial, and incubate in a shaking incubator at 37 °C for 60 min for oximation. After removing the sample, add 50 μL of BSTFA derivatization reagent and 20 μL of n-hexane, along with 10 μL of 10 internal standards (C8/C9/C10/C12/C14/C16/C18/C20/C22/C24, all prepared in chloroform). React at 70 °C for 60 min. After removing the sample, let it stand at room temperature for 30 min, and then perform GC-MS metabolomics analysis.

### 2.4. Gas Chromatography-Mass Spectrometry Analysis Conditions

#### 2.4.1. Chromatographic Conditions

DB-5MS capillary column (30 m × 0.25 mm × 0.25 μm, Agilent J&W Scientific, Folsom, CA, USA), with high-purity helium (purity not less than 99.999%) as the carrier gas, at a flow rate of 1.0 mL/min, and an injection port temperature of 260 °C. Injection volume was 1 μL, with splitless injection, and a solvent delay of 4.8 min. Temperature program: the initial column oven temperature was 60 °C, held for 0.5 min; increased at 8 °C/min to 125 °C; increased at 8 °C/min to 210 °C; increased at 15 °C/min to 270 °C; increased at 20 °C/min to 305 °C, held for 5 min.

#### 2.4.2. Mass Spectrometry Conditions

Electron impact ionization source (EI), ion source temperature 230 °C, quadrupole temperature 150 °C, electron energy 70 eV. The scanning mode was full scan (SCAN), with a mass scan range of *m*/*z* 50–500.

#### 2.4.3. Qualitative and Quantitative Metabolite Analyses

MS-DIAL was used for the qualitative and quantitative analysis of small molecule mass deconvolution based on full scan mode GC-MS data. The raw GC-MS data (.D format) was converted to analysis base file (abf) format using Analysis Base File Converter 4.0.0 software for rapid data retrieval. The data were then imported into MS-DIAL v4.24 software for preprocessing. Algorithms were used to extract ‘model peaks’ from the chromatogram, remove background noise, and achieve compound identification and quantification through retention time, fragment ion mass spectra, and similarity matching with a self-built database. MS-DIAL also provided additional functions required for untargeted metabolomics, such as peak alignment, filtering, and missing value interpolation.

MS-DIAL performs a series of processes on the imported data, including peak detection, peak identification, deconvolution, qualitative analysis, peak alignment, filtering, and missing value interpolation. The final output is a raw data matrix. This three-dimensional matrix includes information such as sample details, the names of substance peaks, retention times, mass-to-charge ratios, and mass spectrometry response intensities (peak areas).

GC-MS qualitative analysis was performed using the LUG database (Untarget database of GC-MS from Lumingbio), which contains 2543 metabolites detectable by GC-MS and is continuously updated. The mass-to-charge ratio range is between 85 and 650, covering various substances including lipids, amino acids, fatty acids, amines, alcohols, sugars, amino sugars, sugar alcohols, sugar acids, organic phosphates, hydroxy acids, aromatics, purines, and sterols. For headspace injection GC-MS experiments studying volatile substances and similar compounds, we used the NIST database (https://webbook.nist.gov/chemistry/ (accessed on 6 September 2024)) for qualitative analysis.

#### 2.4.4. Kyoto Encyclopedia of Genes and Genomes (KEGG) Annotations and Metabolic Pathway Analyses of Differential Metabolites

KEGG is the primary public pathway-related database that includes not only genes but metabolites. Metabolites were mapped to KEGG metabolic pathways for pathway analysis and enrichment analysis. Pathway enrichment analysis identified significantly enriched metabolic pathways or signal transduction pathways in differential metabolites compared with the background. The calculating formula is as follows:

$$P=1-\overset{m-1}{\underset{i=0}{\sum }}\frac{\left(\begin{array}{c} M\\ i \end{array}\right)\left(\begin{array}{c} N-M\\ n-i \end{array}\right)}{\left(\begin{array}{c} N\\ n \end{array}\right)}$$

Here, *N* is the number of all metabolites that with KEGG annotation, *n* is the number of differential metabolites in *N*, *M* is the number of all metabolites annotated to specific pathways, and m is number of differential metabolites in *M*. The calculated *p*-value was gone through FDR Correction, taking FDR ≤ 0.05 as a threshold. Pathways meeting this condition were defined as significantly enriched pathways in differential metabolites.

### 2.5. Data Statistical Analysis

Experimental data were statistically organized using Excel 2010. Analysis of variance (ANOVA) was performed using SPSS (version 22.0, IBM, Armonk, NY, USA). Statistical analysis was conducted to examine the significance of differences in the experimental results, with all data expressed as mean ± standard deviation. Differences between the two groups were considered significant at *p* < 0.05. OPLS-DA and VIP value analyses were performed using Simca 14.1 software.

## 3. Results and Discussion

### 3.1. Effects of Different Shaping Methods and Loading Treatments on Photosynthetic Rate of Plant Functional Leaves

**Figure 1:**
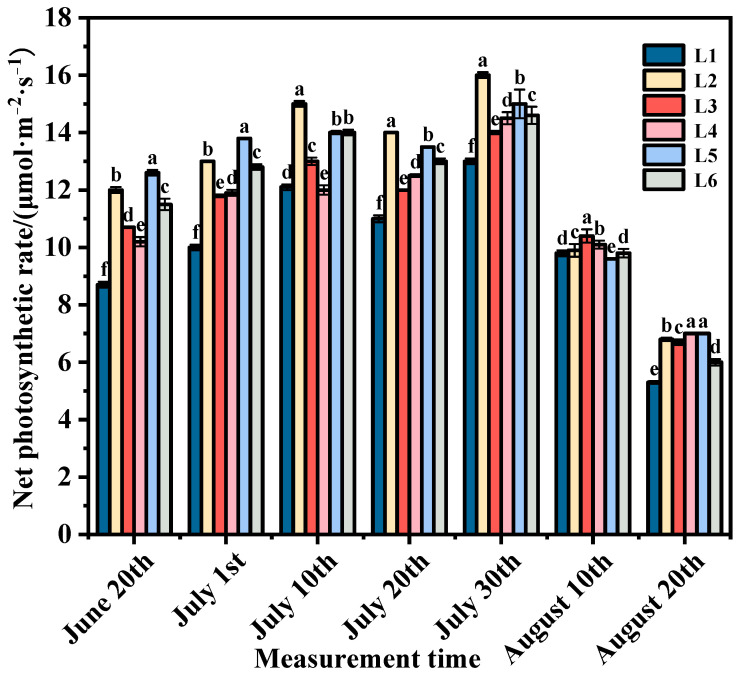
Effects of different shaping methods and loading on the net photosynthetic rate, means with different letters in the same column express significant differences (Duncan’s test *p* < 0.05).

The periodic changes in the net photosynthetic rate of ‘Beibinghong’ grape leaves under different treatments are shown in Figure 1. Through in-depth analysis of these changes, we found that in June and July, the net photosynthetic rate of functional leaves in each group showed a higher level. During this time, the fruit begins to expand rapidly, and the leaves continue to grow. The enhancement of physiological activity may be directly related to the increase in net photosynthetic rate. Due to the rapid development of fruits and the expansion of leaf area, the plant’s utilization efficiency of light energy is improved, which promotes the increase in net photosynthetic rate. In particular, the net photosynthetic rate of leaves peaked in late July, highlighting the importance of this period in the plant’s annual growth cycle. With the advance of the season and the gradual decrease in temperature, the net photosynthetic rate of each treatment group began to show a downward trend. This may be due to the fact that low temperature affects the activity of photosynthetic enzymes and reduces photosynthetic efficiency, resulting in a decrease in net photosynthetic rate [22,23]. This observation is consistent with previous research results and further confirms that environmental temperature is one of the important factors affecting photosynthesis [24,25]. In other growth periods except, August 10, the net photosynthetic rate of L2–L6 treatment was significantly higher than that of L1 treatment, which indicated that the inclined horizontal dracana tree type could significantly increase the net photosynthetic rate of plants compared with the upright tree type with double main tendrine. The net photosynthetic rates at L2 and L5 were significantly higher than those of the control and other treatments. The reason for this is that under the condition of lower load (L2), the plants had vigorous vegetative growth, their leaves became larger and thicker, and the photosynthetic rate of the leaves was higher. As the load increased, the photosynthetic rate gradually rose. The reason lies in that within the reasonable range of load, the increase in load enhanced the source–sink ratio, enabling the photosynthetic potential of source leaves to be further exerted, thereby increasing the photosynthetic rate. These results indicate that the load can affect the photosynthetic level of plants.

### 3.2. Effects of Different Training Systems and Load Treatments on the Chlorophyll Content of Functional Leaves

**Figure 2:**
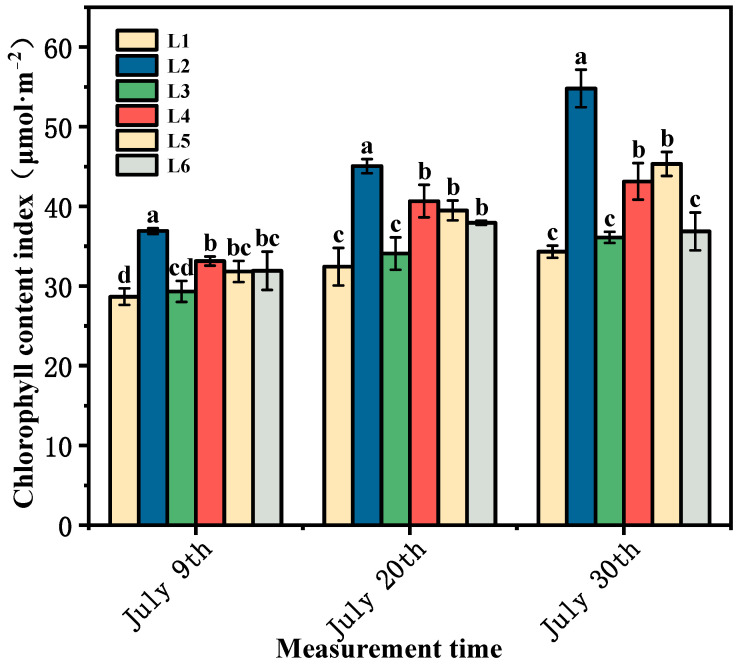
Effects of different shaping methods and loading on chlorophyll content of function leaves, means with different letters in the same column express significant differences (Duncan’s test *p* < 0.05).

As shown in Figure 2, the chlorophyll content of the leaves treated with L2–L6 was higher than that of the leaves treated with1 at different growth stages, and the increase in the chlorophyll content of the leaves treated with L2–L6 compared with L1 treatment was 1.7–28.75% on 9 July; 3.64–38.91% on 20 July; 3.27–59.67% on 30 July. Except for L3, there was a significant difference between the other treatments and the control (*p* < 0.05). These data indicate that the inclined horizontal draconia tree shape contributes to the chlorophyll accumulation in the functional leaves of ‘Beibinghong’ grape. Chlorophyll is the main photosynthetic pigment in plant leaves, and its content is closely related to photosynthesis. Different tree shapes will affect the accumulation of chlorophyll in grape leaves, thus affecting photosynthesis and ultimately affecting grape quality [26]. In addition, different loading treatments also had significant effects on the chlorophyll content of functional leaves of ‘Beibinghong’. L2 treatment was significantly higher than other treatments, followed by L4 and L5 treatments were significantly higher than L3 and L6 treatments. The overall performance was that the lower the load, the higher the chlorophyll content. This result may be due to the fact that with the lower load, plants can allocate more resources (such as nutrients, water, and energy) to physiological processes related to photosynthesis. In addition, in the case of the lower load, the leaves receive more sufficient light, which is not only conducive to chlorophyll synthesis, but also improve the rate of photosynthesis, but also further promote the accumulation of chlorophyll.

### 3.3. Effects of Different Training Systems and Load Treatments on Plant Yield and Fruit Quality

As can be seen from Table 2, the yield of ‘Beibinghong’ L2–L6 treatment gradually increased with the increase in branch retention, in which the yield of L6 treatment was the highest, followed by L5 treatment, but both were higher than the control, indicating that under proper load, the production potential of inclined transverse dragon tree could be higher than that of traditional double main upright tree. The reducing sugar content of fruits treated with L2–L6 was significantly higher than that treated with L1 (*p* < 0.05), and the increase ratio was 10.33–17.50%, but there was no significant difference in reducing sugar content between L2–L6 treatments. Compared with L1, there were significant differences in titrable acid content and sugar–acid ratio in L2–L6 treatment (*p* < 0.05), and significant differences between L4, L5 treatment and L2, L3, and L6 treatment, among which L5 treatment had the lowest titrable acid content. The tannin content of individual treatment is different, but the tannin content of each treatment is not high, and it is in the appropriate range of wine quality requirements. These results show that, compared with the traditional vertical tree with two main tendrils, the tilted horizontal Drucker tree can increase the sugar content of fruits, reduce the titrable acid content, and increase the sugar–acid ratio, which indicates that compared with the traditional tree, the tilted horizontal Drucker tree can improve the wine quality of fruits [27,28,29].

### 3.4. Untargeted Metabolomics Analysis of ‘Beibinghong’ Grapes

#### Volatile Metabolites of ‘Beibinghong’ Grapes

**Figure 3:**
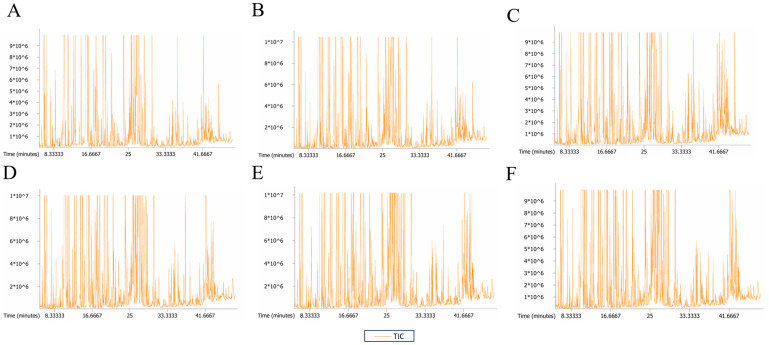
Total Ion Chromatogram (TIC) of ‘Beibinghong’ Grapes under Different Tree Shapes and Load Treatments Detected by GC-MS. (**A**) L1 total ion current. (**B**) L2 total ion current. (**C**) L3 total ion current. (**D**) L4 total ion current. (**E**) L5 total ion current. (**F**) L6 total ion current.

To better understand the differences in volatile metabolites among ‘Beibinghong’ samples under different tree shapes and load levels, we conducted extensive targeted GC-MS volatile metabolite analysis on six groups of ‘Beibinghong’ samples. We used MS-DIAL for small molecule mass deconvolution qualitative and quantitative analysis based on Full Scan mode gas chromatography–mass spectrometry (GC-MS) data, and the NIST database (https://webbook.nist.gov/chemistry/ (accessed on 27 January 2024)) for substance identification. The Base Peak Chromatogram (BPC), which continuously depicts the intensity of the strongest ions at each time point in the chromatogram, is shown in Figure 3.

**Figure 4:**
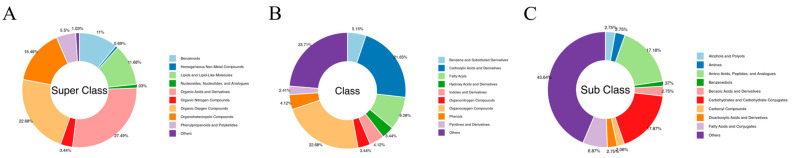
Proportion of each category. (**A**) Super Class. (**B**) Class. (**C**) Sub Class.

Gas chromatography–mass spectrometry (HS-GC-MS) is a common method for separating and quantifying volatile compounds in food [30,31,32]. First, a visual inspection was conducted on the total ion current (TIC) chromatograms of all samples. It was discovered that the instrumental analysis of all samples exhibited strong signals, large peak volumes, and excellent retention time reproducibility (Figure 3). In this study, a total of 291 volatile metabolites were detected and identified. To more intuitively display the classification of these metabolites, we systematically categorized them. Specifically, we selected the nine most abundant classes of metabolites and created a pie chart (Figure 4) based on their Class, Super Class, and Sub Class hierarchical relationships, while the remaining metabolites were uniformly classified as ‘others’. According to the detection results, these metabolites can be further divided into 12 major categories, including: organic acids and derivatives (80 types), organic oxides (66 types), organic cyclic compounds (45 types), lipids and lipid-like molecules (34 types), benzene compounds (32 types), phenylpropanoids and polyketides (16 types), organic nitrogen compounds (10 types), nucleotides and analogs (three types), organic sulfur compounds (one type), alkaloids and derivatives (one type), homogenous non-metal compounds (two types), and nucleosides, hydrocarbons (one type). Under different treatment conditions, the types and contents of metabolites in ‘Beibinghong’ also change, and the proportion of each category is shown in Figure 4. By detecting metabolite classification, we can more clearly understand the effects of different treatments on plant metabolic pathways, and how these effects affect the changes in the physiological state of ‘Beibinghong’. Figure 4 reflects that there are many types of metabolites in grapes, and the metabolites are divided into many categories, such as amino acids, sugars, phenols, flavonoids, tannins, etc., reflecting the complexity and diversity of plant metabolic pathways. According to Figure 4 and Table 3, the metabolite types among the treatment groups are the same, and the difference type should be due to the significant difference in metabolite content.

### 3.5. Multivariate Statistical Analysis of Metabolic Profile Differences in ‘Beibinghong’ Grapes Under Different Load Levels

**Figure 5:**
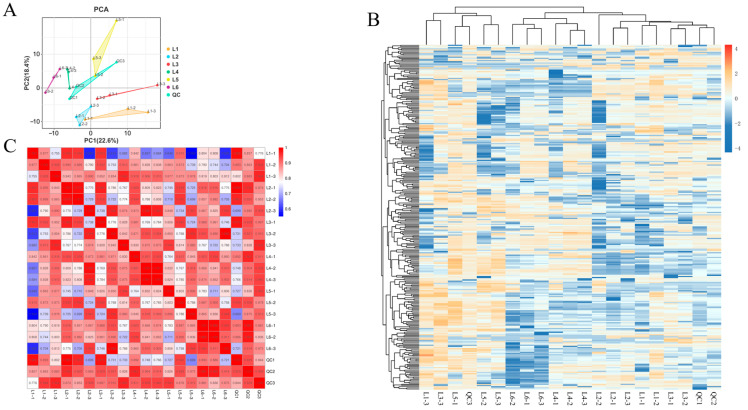
Principal component analysis, heat map analysis, and correlation analysis of relative differences in metabolite content of ‘Beibinghong’ grapes from six treatments. (**A**) Principal component analysis of metabolites of six samples. (**B**) Clustering heat map analysis of metabolites of six treated ‘Northern Ice Red’ grape samples. The color indicates the accumulation level of each metabolite, with higher values from low (blue) to high (red) and darker colors indicating that the substance is high in each subgroup. (**C**) Correlation analysis of metabolites in six treated ‘Beibonghong’ grape samples. The color indicates the level of accumulation of each metabolite, with higher values from low (blue) to high (red) and darker colors indicating a stronger correlation between the two samples.

PCA analysis uses several principal components to reflect the characteristics of metabolomics multidimensional data and to reveal the internal structure of multivariate data through these components [33,34]. Therefore, we can observe the differences between different groups through the PCA plot. By performing unsupervised multivariate statistical analysis (PCA) on the samples, we determined the degree of variation between different treatments of ‘Beibinghong’ grape samples, between groups, and within groups (Figure 5A). The contribution rate of PC1 is 22.6%, and PC2 is 18.4%. The six groups of samples show a separation trend on the two-dimensional plot, with no outliers, and the grape samples from the same treatment cluster well. The PCA results indicate that there are significant differences in metabolites among the six groups of samples. In the principal component analysis plot, the quality control (QC) samples, which are mixtures of grape samples, are projected into the same region; some even overlap, indicating that they have similar metabolic profiles, and our analysis is stable and reproducible.

The clustering heatmap also significantly divided the samples into six groups (as shown in Figure 5B), which is consistent with the PCA analysis. Therefore, both principal component analysis and clustering analysis indicate that the ‘Beibinghong’ grapes under these six different treatment conditions have distinct metabolic profiles. These differences reveal that different cultivation modes strongly influence the metabolites of ‘Beibinghong’ grapes. Furthermore, the correlation analysis (Figure 5C) shows that the clustering effect among the samples is also good, with a correlation coefficient (r) greater than 0.8 between samples. This indicates that our analysis is highly reproducible and demonstrates a high degree of reliability in showing the differences between ‘Beibinghong’ grape samples under different treatments.

#### 3.5.1. Key Compounds Associated with ‘Beibinghong’ Flavor Difference

Based on the metabolic profile differences revealed by the above PCA and clustering analyses, we further conducted an in-depth differential analysis to specifically identify and quantify the changes in metabolites of ‘Beibinghong’ grapes under different load levels, aiming to reveal the specific association between load changes and grape metabolites. Orthogonal Partial Least Squares Discriminant Analysis (OPLS-DA) is a derivative algorithm of PLS-DA. Compared with PLS-DA, OPLS-DA combines Orthogonal Signal Correction (OSC) and PLS-DA methods [35,36], which can decompose the X matrix information into two types: related and unrelated to Y. By removing unrelated differences, the relevant information is concentrated in the first predictive component. Subsequent model validation and differential metabolite screening are analyzed using OPLS-DA results. This method is a supervised pattern recognition multivariate statistical analysis method that can effectively eliminate irrelevant influences to maximize the observation of inter-group differential metabolites [37].

**Figure 6:**
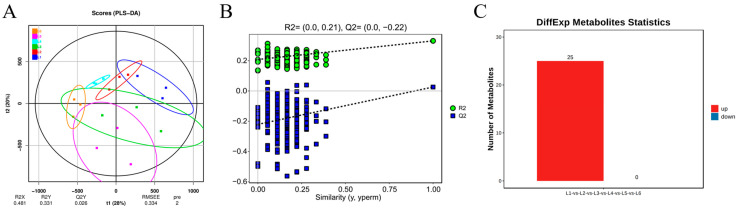
OPLS-DA analyses of ‘Beibinghong’ samples under different treatments. (**A**,**B**) Sample OPLS-DA analysis. (**C**) Differential metabolite number plot.

OPLS-DA can be evaluated using R_2_X, R_2_Y, Q2, and OPLS-DA score plots to assess the classification effectiveness of the model. The VIP (Variable Importance for the Projection) value indicates the importance of variables (feature peaks) in explaining the X dataset and their association with the Y dataset (Figure 6). In the comparison of sample groups, Q2 values are all greater than 0.9, indicating that the model is excellent and performs better than the PCA model. Permutation validation of OPLS-DA (n = 200, i.e., 200 permutation experiments) showed that the R2 and Q2 of the original model are greater than those of the Y-permuted model, indicating that the model’s predictive results are reliable. Variables with VIP > 1 can be used as one of the criteria for screening potential biomarkers. Among the treatment group and the control group, 25 differential metabolites with VIP values ≥ 1 were screened out. The substances that were mainly up-regulated included 4-aminobutyric acid, trehalose, Mannitol, Threitol and glucuronic acid. Except for trehalose, the other four metabolites were the main substances contributing to the aroma differences between L2–L6 treatment and L1 control (Table 4). These metabolites may be key to the aroma differences caused by different load levels.

**Figure 7:**
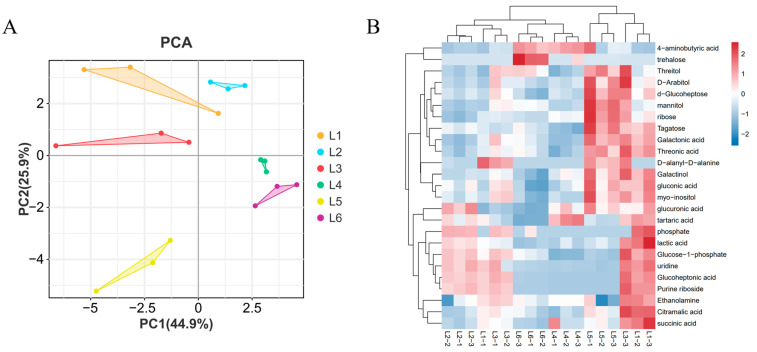
Principal Component Analysis and Heatmap Analysis of Relative Differences in Metabolite Content of ‘Beibinghong’ Grapes under Six Treatments. (**A**) Principal Component Analysis (**B**) Heatmap Analysis.

**Figure 8:**
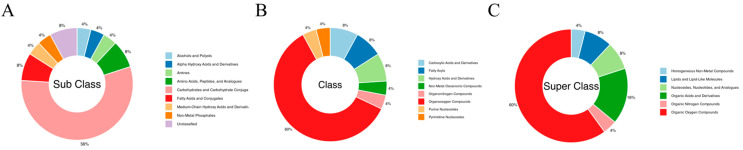
Pie charts of 25 metabolite levels at each level. (**A**) Sub Class. (**B**) Class. (**C**) Super Class.

PCA and clustering heatmap analysis of the 25 screened metabolites showed (Figure 7) that the volatile metabolite content of different groups varied significantly. The 25 screened metabolites were classified, and the nine most abundant classes were selected to create pie charts based on Class, Super Class, and Sub Class (Figure 8), with other classes combined into ‘others’. The classification pie chart is shown below. According to the detection results, the metabolites were divided into twelve major categories, including fifteen organic oxides, two lipids and lipid-like molecules, four organic acids and derivatives, two nucleosides, nucleotides, and analogs, one homogenous non-metal compound, and one organic nitrogen compound. The proportions of each category according to Super Class classification are shown in Table 5. The metabolites involved in different load levels are diverse, including mannitol, tartaric acid, myo-inositol, etc. These metabolites hold significant positions in the model. Compounds with high VIP values significantly impact the flavor of grape berries. Among the 25 compounds with VIP values greater than 1, most are alcohols, acids, and sugars. Alcohols are crucial components of grape aroma and significantly influence the taste and flavor of wine, adding richness and fullness. The compound with the highest VIP value is mannitol, which primarily acts as an osmotic regulator and sweetener in grape berries. It helps the berries adapt to different growing environments, maintain normal cellular functions, and enhance the taste and flavor of the fruit [38]. Acids not only directly affect the taste and aroma of wine grapes but also undergo various chemical reactions during wine processing, significantly influencing the final flavor of the wine [39,40]. Among these, tartaric acid, also known as 2,3-dihydroxybutanedioic acid, with the molecular formula C_4_H_6_O_6_, contains two chiral carbon atoms, producing three optical isomers: levorotatory, dextrorotatory, and meso forms [41]. Tartaric acid is one of the characteristic acids in grape berries and plays a crucial role in defining grape flavor. Among the organic acids affecting grape flavor, tartaric acid is the most abundant [42]. Generally, in the absence of external additives, the tartaric acid in wine comes entirely from grapes, indicating a direct relationship between the tartaric acid content in grape berries and in wine. During processing, tartaric acid from grapes easily integrates into wine, helping to maintain acidity, lower pH, inhibit bacterial growth, and preserve long-term freshness. It is crucial for strengthening the structure of grape berries, enhancing their flavor characteristics, and preserving their color integrity. Other organic acids such as succinic acid, lactic acid, and citric acid interact with aromatic substances, enhancing or modifying the aroma of wine grapes and thus influencing the flavor of the wine. Sugars are one of the main flavor components in grape berries [43]. They not only provide sweetness but also serve as raw materials for yeast fermentation, producing alcohol and carbon dioxide.

**Figure 9:**
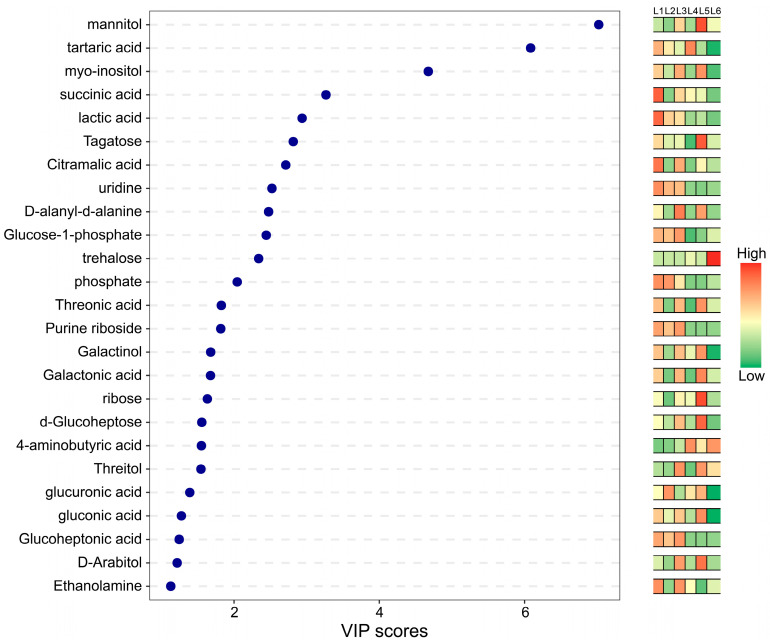
Differential metabolite VIP values and heatmap (horizontal coordinates indicate the magnitude of the VIP value of the substance, the color of the heatmap on the right hand side ranges from low (green) to high (red) the higher the value, the higher the content).

The variation in VIP values can be explained by differences in the content, stability, and correlation of these metabolites with diseases or physiological states in the organism [16]. In model prediction or classification, the importance of these metabolites increases, meaning that metabolites with higher VIP values have a greater impact on the flavor of wine grapes, the VIP values for each key metabolite are shown in Table 6. The VIP score graph is shown in Figure 9.

#### 3.5.2. KEGG Classification and Enrichment Analysis of Key Metabolites

The KEGG differential metabolite enrichment score plot provides us with a comprehensive perspective to understand the enrichment degree and statistical significance of different metabolic pathways in differential metabolites. This information is crucial for revealing the mechanisms of biological processes, discovering new biomarkers, and guiding subsequent experimental designs [44]. We mapped the 25 screened metabolites to the KEGG database, and the results showed that most metabolites were enriched in the ‘metabolic pathways’ of secondary metabolites, which is consistent with expectations. Additionally, some metabolites were enriched in ‘organismal systems’, ‘environmental information processing’, ‘cellular processes’, and ‘human diseases’, indicating that some metabolites may have potential impacts on the environment and health. Among the 25 metabolites, a few were classified into ‘pentose and glucuronate interconversions’, ‘microbial metabolism in diverse environments’, ‘nucleotide sugar biosynthesis’, ‘glucagon signaling pathway’, and ‘cAMP signaling pathway’, as well as pathways related to human diseases, suggesting that these metabolites may play roles in microbial metabolism and signal transduction.

**Figure 10:**
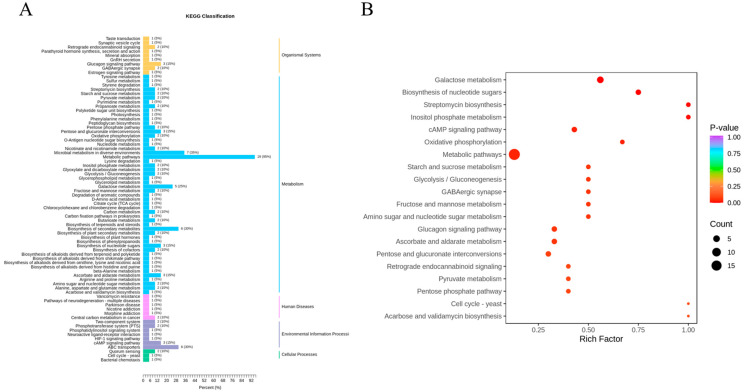
Metabolite KEGG differential enrichment classification plot and KEGG differential abundance score plot. (**A**) Metabolite KEGG differential enrichment classification plot. (**B**) KEGG differential abundance score plot.

The KEGG differential metabolite enrichment score plot (Figure 10) provides us with a visual representation of the enrichment degree of differential metabolites in various metabolic pathways. In the plot, each point represents a specific metabolic pathway, with its position determined by the enrichment score (Rich Factor), and the size and color of the points reflecting the number of differential metabolites and their statistical significance (*p*-value), respectively. From the enrichment scores (Figure 10B), pathways with higher scores, such as ‘Metabolic pathways’ and ‘Starch and sucrose metabolism’, indicate a higher proportion of differential metabolites in these pathways, suggesting that these pathways are more concentrated with differential metabolites compared to the entire metabolite pool. This implies that these pathways may play important roles in the cultivation modes of ‘Beibinghong’ grapes. Metabolites enriched in the ‘Starch and sucrose metabolism’ pathway not only affect the accumulation of sugars but may also indirectly influence the acidity of the fruit. Although acidity is primarily determined by organic acid metabolism, the accumulation of sugars can affect the balance of sweetness and acidity in the fruit, thereby influencing the overall taste. Metabolites in this pathway may also be involved in the synthesis of aromatic compounds, impacting the flavor of wine grapes and consequently the flavor of the wine. By combining enrichment scores and *p*-values, we can identify pathways that are both significantly enriched and statistically important, such as ‘Metabolic pathways’ and ‘Starch and sucrose metabolism’, which will be the focus of our subsequent research.

### 3.6. Multivariate Statistical Analysis of Metabolic Profile Differences in ‘Beibinghong’ Grapes Under Different Cultivation Trellis Systems

**Figure 11:**
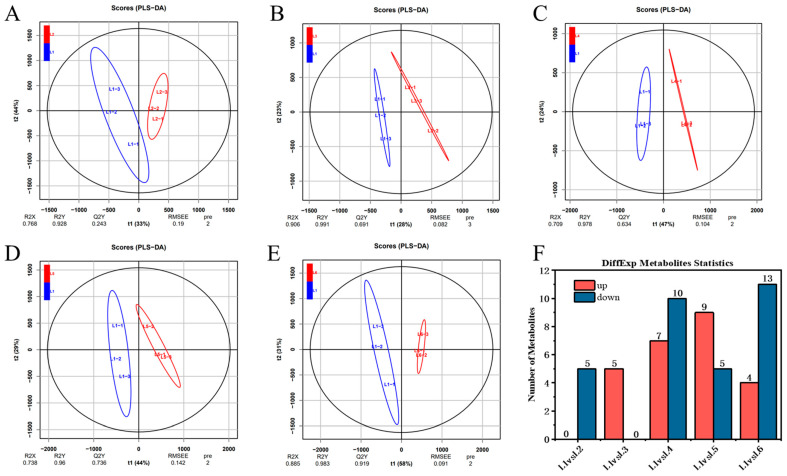
OPLS-DA Analysis and Differential Metabolite Count Statistics for Different Treatments. (**A**) Opls-da two-dimensional score plot of L1 vs. L2. (**B**) Opls-da two-dimensional score plot of L1 vs. L3. (**C**) Opls-da two-dimensional score plot of L1 vs. L4. (**D**) Opls-da two-dimensional score plot of L1 vs. L5. (**E**) Opls-da two-dimensional score plot of L1 vs. L6. (**F**) Differential metabolite count statistics for different treatments. The horizontal axis represents each differential comparison group, and the vertical axis represents the number of differential metabolites in each comparison group. Red indicates the number of upregulated differential metabolites in the latter compared to the former in the comparison group, while blue indicates the number of downregulated differential metabolites.

To investigate the impact of different cultivation trellis systems on the metabolic profile of ‘Beibinghong’ grapes and accurately characterize it, we used multivariate statistical analysis methods. We used the double main vine upright tree shape L1 as the control group and set the inclined horizontal dragon trunk L2 to L6 as the treatment groups, with L1 vs. L2, L1 vs. L3, L1 vs. L4, L1 vs. L5, and L1 vs. L6 as the groups. To avoid false positives resulting from using only one statistical analysis method, we further screened differential metabolites based on VIP ≥ 1 and *t*-test *p* < 0.05. The number of differential metabolites is shown in Figure 11F.

Through the OPLA-DA score map (Figure 11), the degree of difference between different groups can be intuitively displayed. The abscissa in the score plot represents the predicted principal components, reflecting the main differences between groups; The ordinates represent orthogonal principal components, reflecting differences within groups. Through the score map, it can be clearly seen that there are significant differences between different groups under different treatments. Between L1 and L2, five metabolites were screened (all downregulated); between L1 and L3, five metabolites were screened (all upregulated); between L1 and L4, 17 metabolites were screened (seven upregulated, ten downregulated); between L1 and L5, 14 metabolites were screened (nine upregulated, five downregulated); and between L1 and L6, 17 metabolites were screened (four upregulated, thirteen downregulated). In these group comparisons, the upregulated metabolites indicate their greater importance under specific cultivation conditions, such as enhanced energy metabolism, activation of defense mechanisms, or strengthening of specific metabolic pathways [45]. The downregulated metabolites reflect a reduced demand or inhibition of these metabolic pathways under specific cultivation conditions. It is worth noting that the small difference in metabolite content between L1 and L2 and L3 suggests that metabolite differences are not as large in the traditional two-main-vine upright form with relatively stable production or degradation rates at loadings of 12–14, whereas in highly loaded grapes, the differences in compound content are usually greater, which may be due to the fact that high loadings result in greater physiological stresses on the fruiting trees, which alters the synthesis of compounds and metabolite partitioning. As shown in Table 7, the impact of different cultivation trellis systems on the grape metabolic profile may involve multiple aspects, including energy metabolism, osmotic regulation, defense mechanisms, and fruit quality. The upregulation or downregulation of metabolites reflects the plant’s metabolic adaptability and adjustment under different environmental conditions [46]. For example, glucose-1-phosphate, mannitol, and trehalose play roles in energy storage, osmotic regulation, and signal transduction in plants. Their changes in different treatment groups may indicate differences in energy metabolism and osmotic regulation. Succinic acid, tartaric acid, and citric acid participate in various metabolic pathways in plants, such as the tricarboxylic acid cycle, and significantly affect fruit flavor [47,48,49]. Their upregulation or downregulation may reflect changes in energy metabolism and fruit quality under different cultivation conditions. Amino acids such as glycine, 4-aminobutyric acid, and beta-alanine are the basic building blocks of proteins and participate in various metabolic pathways in plants [50]. Their differences may reflect the activity of protein synthesis, degradation, and specific metabolic pathways. Comparing the double main vine upright tree shape with the inclined horizontal dragon trunk cultivation systems, overall, the metabolite levels are downregulated. Therefore, from the perspective of volatile metabolites, the inclined horizontal trellis system is more suitable for cultivation as it is more conducive to the accumulation of volatile compounds and the formation of fruit quality. In future research, we need to pay more attention to metabolic pathways related to fruit quality and aroma, such as the tricarboxylic acid cycle as well as the sugar metabolism pathway.

## 4. Conclusions

This study found that in different growth stages, the inclined horizontal trunk tree form and the upright tree form with two main branches could significantly increase the chlorophyll content and net photosynthetic rate of plant leaves compared with each other. The reducing sugar content of fruit was increased (10.33–17.50%) and the ratio of sugar to acid was increased. It was found that 25 different metabolites of 12 kinds of substances, including lipids, organic acids and organic compounds, were the key substances that caused the differences among different treatments. These substances were mainly concentrated in the metabolic pathways of starch and sucrose in the KEGG database. The overall metabolite level of inclined horizontal dracaena tree was upregulated compared with that of double main tendracaena tree. Therefore, from the point of view of volatile metabolites, it is more suitable to choose the inclined horizontal dracakes in cultivation, because it is more conducive to the accumulation of volatile compounds and the formation of fruit quality.

It was found that the treatment with nine branches and eighteen ears of fruit performed well in terms of fruit yield, reducing sugar content, titrable acid content and sugar–acid ratio. It was a suitable loading treatment. Therefore, it is suggested that the inclined horizontal dragon stem tree is more suitable for planting the northern ice red grape, and it is more reasonable to cultivate nine fruit branches and eighteen ears of fruit in Jilin area, and the yield is controlled at 1000 kg/667 m^2^.

The research results provide theoretical support and practical guidance for optimizing the grape cultivation management of ‘Beibinghong’, improving fruit quality and enhancing market competitiveness. Future studies can further explore the specific regulatory mechanisms of different cultivation modes on grape metabolic pathways and the role of key metabolites in the formation of fruit flavor.

## Figures and Tables

**Table 1:** Experimental treatment.

Treatment	Shape	Number of Branches/pc	Fruit Retention/Ear	Total Load/Spike
L1	Double main vine upright tree form	25	1~2	30~40
L2	Tilting horizontal dragon stem	6	2	12
L3	Tilting horizontal dragon stem	7	2	14
L4	Tilting horizontal dragon stem	8	2	16
L5	Tilting horizontal dragon stem	9	2	18
L6	Tilting horizontal dragon stem	10	2	20

**Table 2:** The effects of different shaping methods and loading on production and fruit quality of ‘Beibinghong’ grape.

Treatment	Production/(kg·ha^−1^)	Soluble Sugar (g·L^−1^)	Titratable Acidity (g·L^−1^)	Tanins/(mg·g^−1^)	The Ratio of Sugar and Acid	Single Grain Weight (g)
L1	14,218 ± 351 abc	152.00 ± 10.00 b	16.00 ± 0.07 a	0.12 ± 0.00 ab	9.50 ± 0.05 c	0.94 ± 0.10 c
L2	11,935 ± 194 c	170.30 ± 7.40 a	14.90 ± 0.11 b	0.12 ± 0.01 bc	11.43 ± 0.79 b	1.06 ± 0.07 abc
L3	13,136 ± 203 bc	174.10 ± 3.20 a	14.90 ± 0.08 b	0.13 ± 0.10 a	11.68 ± 0.57 b	1.08 ± 0.05 abc
L4	12,816 ± 152 bc	167.70 ± 11.00 a	14.20 ± 0.06 c	0.10 ± 0.10 c	11.81 ± 0.49 ab	1.10 ± 0.11 ab
L5	14,859 ± 406 ab	169.60 ± 3.70 a	14.10 ± 0.07 c	0.13 ± 0.01 a	12.03 ± 0.76 a	1.14 ± 0.04 a
L6	16,020 ± 798 a	178.60 ± 11.30 a	15.30 ± 0.07 b	0.11 ± 0.00 bc	11.67 ± 0.43 b	0.98 ± 0.01 bc

Note: Data are presented as mean ± standard deviation, different letters after the same column of numbers in the table indicate that the difference reaches a significant level (*p* < 0.05).

**Table 3:** Summary of total metabolite classification.

Super Class	Count	Percent
Benzenoids	32	11%
Homogeneous Non-Metal Compounds	2	0.69%
Lipids and Lipid-Like Molecules	34	11.68%
Nucleosides, Nucleotides, and Analogs	3	1.03%
Organic Acids and Derivatives	80	27.49%
Organic Nitrogen Compounds	10	3.44%
Organic Oxygen Compounds	66	22.68%
Organoheterocyclic Compounds	45	15.46%
Phenylpropanoids and Polyketides	16	5.5%
Others	3	1.03%

**Table 4:** Differential metabolites of ‘Beibinghong’ under different treatments.

Metabolites	L1	L2	L3	L4	L5	L6
mannitol	(5.9 ± 1.2) × 10^4^ d	(4.6 ± 0.3) × 10^4^ f	(8.8 ± 1.5) × 10^4^ b	(5.2 ± 0.3) × 10^4^ e	(1.3 ± 0.2) × 10^5^ a	(7.1 ± 0.2) × 10^4^ c
tartaric acid	(2.0 ± 0.3) × 10^5^ b	(1.7 ± 0.2) × 10^5^ c	(1.5 ± 0.4) × 10^5^ d	(2.2 ± 0.2) × 10^5^ a	(1.3 ± 0.08) × 10^5^ e	(0.9 ± 0.02) × 10^5^ f
myo-inositol	(1.0 ± 0.2) × 10^5^ c	(0.8 ± 0.05) × 10^5^ d	(1.1 ± 0.09) × 10^5^ b	(0.8 ± 0.03) × 10^5^ e	(1.1 ± 0.2) × 10^5^ a	(0.7 ± 0.08) × 10^5^ f
succinic acid	(1.2 ± 0.2) × 10^5^ a	(0.7 ± 0.04) × 10^5^ e	(1.0 ± 0.2) × 10^5^ b	(0.9 ± 0.3) × 10^5^ c	(0.9 ± 0.08) × 10^5^ d	(0.7 ± 0.03) × 10^5^ f
lactic acid	(5.5 ± 1.4) × 10^4^ a	(4.5 ± 0.1) × 10^4^ b	(4.3 ± 0.9) × 10^4^ c	(3.3 ± 0.3) × 10^4^ e	(3.5 ± 0.2) × 10^4^ d	(3.0 ± 0.1) × 10^4^ f
tagatose	(2.8 ± 0.6) × 10^4^ b	(2.3 ± 0.1) × 10^4^ d	(2.3 ± 0.6) × 10^4^ c	(1.5 ± 0.2) × 10^4^ f	(3.7 ± 0.5) × 10^4^ a	(2.2 ± 0.3) × 10^4^ e
citramalic acid	(6.6 ± 1.0) × 10^4^ a	(4.3 ± 0.3) × 10^4^ e	(6.1 ± 1.3) × 10^4^ b	(4.2 ± 0.3) × 10^4^ f	(5.3 ± 0.4) × 10^4^ c	(4.6 ± 0.5) × 10^4^ d
uridine	(1.6 ± 0.4) × 10^4^ a	(1.3 ± 0.2) × 10^4^ b	(1.2 ± 0.8) × 10^4^ c	(8.9 ± 0.9) × 10^2^ e	(3.3 ± 2.2) × 10^2^ f	(1.4 ± 0.2) × 10^3^ d
D-alanyl-D-alanine	(5.2 ± 0.9) × 10^3^ c	(3.6 ± 0.6) × 10^2^ d	(1.2 ± 0.2) × 10^4^ a	0 ± 0 e	(1.0 ± 0.2) × 10^4^ b	0 ± 0 e
glucose-1-phosphate	(4.2 ± 0.8) × 10^4^ b	(4.0 ± 0.2) × 10^4^ c	(4.6 ± 0.9) × 10^4^ a	(1.7 ± 0.08) × 10^4^ f	(2.1 ± 0.1) × 10^4^ e	(2.9 ± 0.3) × 10^4^ d
trehalose	(1.1 ± 0.06) × 10^2^ e	(1.1 ± 0.9) × 10^2^ f	(1.3 ± 0.01) × 10^4^ d	(2.6 ± 0.5) × 10^3^ b	(6.0 ± 0.8) × 10^3^ c	(2.1 ± 0.3) × 10^4^ a
phosphate	(1.3 ± 0.1) × 10^4^ a	(1.2 ± 0.04) × 10^4^ b	(7.4 ± 0.3) × 10^3^ c	(0.5 ± 0.06) × 10^2^ f	(0.6 ± 0.1) × 10^2^ e	(2.7 ± 0.3) × 10^3^ d
threonic acid	(1.5 ± 0.3) × 10^4^ c	(1.0 ± 0.08) × 10^4^ e	(1.5 ± 0.3) × 10^4^ b	(0.9 ± 0.1) × 10^4^ f	(1.6 ± 0.1) × 10^4^ a	(1.2 ± 0.1) × 10^4^ d
purine riboside	(7.5 ± 1.6) × 10^3^ b	(6.1 ± 0.7) × 10^3^ c	(7.8 ± 1.6) × 10^3^ a	0 ± 0 e	0 ± 0 e	(1.5 ± 0.2) × 10^2^ d
galactinol	(2.0 ± 0.4) × 10^4^ c	(1.6 ± 0.06) × 10^4^ e	(2.1 ± 0.4) × 10^4^ b	(1.8 ± 0.1) × 10^4^ d	(2.2 ± 0.3) × 10^4^ a	(1.4 ± 0.1) × 10^4^ f
galactonic acid	(8.5 ± 1.5) × 10^3^ c	(5.2 ± 0.3) × 10^3^ e	(9.0 ± 1.6) × 10^3^ b	(5.1 ± 0.2) × 10^3^ f	(1.0 ± 0.1) × 10^4^ a	(6.7 ± 0.6) × 10^3^ d
ribose	(7.4 ± 0.9) × 10^3^ c	(5.0 ± 0.3) × 10^3^ f	(7.9 ± 2.2) × 10^3^ b	(7.3 ± 0.3) × 10^3^ d	(1.2 ± 0.1) × 10^4^ a	(6.0 ± 0.2) × 10^3^ e
d-Glucoheptose	(6.6 ± 1.4) × 10^3^ c	(5.5 ± 0.4) × 10^3^ d	(8.0 ± 1.6) × 10^3^ b	(5.4 ± 0.4) × 10^3^ e	(9.7 ± 1.4) × 10^3^ a	(4.5 ± 0.9) × 10^3^ f
4-aminobutyric acid	(2.2 ± 0.4) × 10^2^ f	(4.7 ± 0.5) × 10^2^ e	(2.0 ± 0.3) × 10^3^ d	(7.1 ± 0.8) × 10^3^ a	(4.2 ± 0.4) × 10^3^ c	(6.9 ± 1.0) × 10^3^ b
threitol	(5.2 ± 1.1) × 10^3^ d	(4.8 ± 0.3) × 10^3^ e	(8.4 ± 1.5) × 10^3^ a	(4.4 ± 0.5) × 10^3^ f	(8.3 ± 0.9) × 10^3^ b	(6.9 ± 0.6) × 10^3^ c
glucuronic acid	(9.1 ± 2.0) × 10^4^ d	(1.1 ± 0.7) × 10^5^ a	(8.1 ± 1.0) × 10^4^ e	(9.5 ± 0.8) × 10^4^ c	(1.0 ± 0.1) × 10^5^ b	(6.8 ± 0.2) × 10^4^ f
gluconic acid	(1.2 ± 0.2) × 10^4^ c	(1.1 ± 0.08) × 10^4^ d	(1.2 ± 0.4) × 10^4^ b	(1.0 ± 0.09) × 10^4^ e	(1.3 ± 0.2) × 10^4^ a	(0.8 ± 0.1) × 10^4^ f
glucoheptonic acid	(3.7 ± 0.7) × 10^3^ b	(3.1 ± 0.2) × 10^3^ c	(4.0 ± 1.0) × 10^3^ a	(1.9 ± 0.04) × 10^2^ e	(1.2 ± 0.2) × 10^2^ f	(2.9 ± 0.4) × 10^2^ d
D-Arabitol	(4.2 ± 0.9) × 10^3^ c	(3.6 ± 0.2) × 10^3^ f	(5.8 ± 1.8) × 10^3^ b	(3.8 ± 0.2) × 10^3^ d	(6.3 ± 1.2) × 10^3^ a	(3.7 ± 0.4) × 10^3^ e
ethanolamine	(1.6 ± 0.3) × 10^4^ a	(7.8 ± 4.7) × 10^3^ e	(1.5 ± 2.6) × 10^4^ b	(1.1 ± 0.02) × 10^4^ c	(6.5 ± 1.6) × 10^3^ f	(1.0 ± 0.2) × 10^4^ d

Note: Data are presented as mean ± standard deviation, different letters after the same column of numbers in the table indicate that the difference reaches a significant level (*p* < 0.05).

**Table 5:** Summary of classification of differential metabolites.

Super Class	Count	Percent
Homogeneous Non-Metal Compounds	1	4%
Lipids and Lipid-Like Molecules	2	8%
Nucleosides, Nucleotides, and Analogs	2	8%
Organic Acids and Derivatives	4	16%
Organic Nitrogen Compounds	1	4%
Organic Oxygen Compounds	15	60%

**Table 6:** Analysis of VIP Values of Volatile Aroma Compounds in ‘Beibinghong’ Grapes under Different Treatments.

Metabolites	L1-vs.-L2-vs.-L3-vs.-L4-vs.-L5-vs.-L6
mannitol	7.021236
tartaric acid	6.08327
myo-inositol	4.674105
succinic acid	3.26552
lactic acid	2.937142
tagatose	2.815011
citramalic acid	2.71205
uridine	2.521777
D-alanyl-D-alanine	2.475674
glucose-1-phosphate	2.443104
trehalose	2.338954
phosphate	2.043574
threonic acid	1.82481
purine riboside	1.817063
galactinol	1.678221
galactonic acid	1.675797
ribose	1.632572
d-Glucoheptose	1.555828
4-aminobutyric acid	1.550444
threitol	1.544151
glucuronic acid	1.39043
gluconic acid	1.275001
glucoheptonic acid	1.24461
D-Arabitol	1.216539
ethanolamine	1.128745

**Table 7:** Differential metabolites between the L1 group and the other 5 groups.

L1 vs. L2	L1 vs. L3	L1 vs. L4	L1 vs. L5	L1 vs. L6
Succinic acid (down)	Oxoproline (up)	Glycerol (up)	Tartaric acid (down)	Glycerol (up)
Citramalic acid (down)	Threitol (up)	Glucose-1-phosphate (down)	Mannitol (up)	Tartaric acid (down)
Threonic acid (down)	beta-Alanine (up)	Citramalic acid (down)	Glucose-1-phosphate (down)	Succinic acid (down)
Galactonic acid (down)	4-aminobutyric acid (up)	Uridine (down)	Uridine (down)	Citric acid (down)
Ribose (down)	Glycine (up)	Tagatose (down)	Purine riboside (down)	Lactic acid (down)
		Purine riboside (down)	Ribose (up)	Trehalose (up)
		4-aminobutyric acid (down)	Glucoheptonic acid (down)	Citramalic acid (down)
		Threonic acid (down)	3,6-Anhydro-D-galactose (up)	Uridine (down)
		Ethanolamine (down)	Threitol (up)	Purine riboside (down)
		Glucoheptonic acid (down)	6-deoxy-D-glucose (up)	4-aminobutyric acid (up)
		Galactonic acid (down)	Gallic acid (up)	Galactinol (down)
		Dehydroascorbic Acid (up)	Gluconic lactone (up)	Ethanolamine (down)
		1-Methylhydantoin (down)	Dehydroascorbic Acid (up)	1-Methylhydantoin (down)
		Glycine (up)	3,4-dihydroxycinnamic acid (up)	Glucoheptonic acid (down)
		D-(glycerol 1-phosphate) (up)		Adenine (up)
		Adenine (up)		
		Caffeic acid (up)		

## Data Availability

The original contributions presented in this study are included in the article. Further inquiries can be directed to the corresponding author.
